# Valvular heart disease in patients with cardiac amyloidosis

**DOI:** 10.1007/s10741-023-10350-1

**Published:** 2023-09-22

**Authors:** Alberto Aimo, Lara Camerini, Iacopo Fabiani, Paolo Morfino, Giorgia Panichella, Andrea Barison, Angela Pucci, Vincenzo Castiglione, Giuseppe Vergaro, Gianfranco Sinagra, Michele Emdin

**Affiliations:** 1 Interdisciplinary Center for Health Sciences, Scuola Superiore Sant’Anna, Piazza Martiri della Libertà 33, 56127 Pisa, Italy; 2 Cardiology Division, Fondazione Toscana Gabriele Monasterio, Pisa, Italy; 3 Histopathology Department, University Hospital of Pisa, Pisa, Italy; 4 Center for Diagnosis and Treatment of Cardiomyopathies, Cardiovascular Department, Azienda Sanitaria Universitaria Giuliano-Isontina (ASUGI) and University of Trieste, Trieste, Italy

**Keywords:** Cardiac amyloidosis, Valve disease, Cardiomyopathy, Aortic valve, Mitral valve

## Abstract

Cardiac amyloidosis (CA) is an underdiagnosed condition caused by the deposition of misfolded proteins, namely immunoglobulin light chains and transthyretin, in the extracellular spaces of the heart. Any cardiovascular structure can be affected by amyloid infiltration, including the valves. Amyloid accumulation within the cardiac valves may lead to their structural and functional impairment, with a profound impact on patients’ prognosis and quality of life. The most common forms of valvular disease in CA are aortic stenosis (AS), mitral regurgitation (MR), and tricuspid regurgitation (TR). CA and AS share similar risk factors, disease mechanisms, and remodeling patterns, which make their diagnosis particularly challenging. Patients with both CA and AS experience worse outcomes than CA or AS alone, and transcatheter aortic valve replacement may represent a useful therapeutic strategy in this population. Data on MR and TR are quite limited and mainly coming from case reports or small series. This review paper will summarize our current understanding on the epidemiology, disease mechanisms, echocardiographic features, clinical implications, and therapeutic options of AS, MR, and TR in patients with CA.

Cardiac amyloidosis is increasingly recognized as an underdiagnosed condition. Its estimated prevalence has increased from 9 to 16 cases per million before 2010 to 40 to 58 cases per million after 2010 [1], following the introduction of a non-invasive diagnostic algorithm, a greater disease awareness and the availability of specific therapeutic options [2–4]. Cardiac amyloidosis (CA) is caused by the extracellular deposition of insoluble fibrils composed of misfolded proteins in the myocardium. Amyloid light-chain (AL) amyloidosis is a plasma-cell disorder characterized by tissue accumulation of free light chains produced by a clonal plasma cell, whereas amyloid transthyretin (ATTR) amyloidosis is caused by the accumulation of either wild-type or mutated (“variant”) TTR (ATTRwt or ATTRv, respectively) [5]. The expansion of the extracellular space due to amyloid deposition leads to myocardial stiffness and diastolic dysfunction [6]. Amyloid may also accumulate in all valvular structures, which are sometimes affected by other disease processes (e.g., fibrocalcific remodeling of the aortic valve). Valvular heart disease (VHD) may affect the clinical manifestations of CA, impact on patient outcomes, and change the therapeutic approach.

A growing interest towards VHD has prompted research in the context of CA. To date, VHD accounts as a red flag for CA suspicion, which could be properly screened by echocardiography. Moreover, valvular involvement determines a significant hemodynamic load on myocardium, with a clearly negative impact on exercise capacity and health.

This review paper will focus on the most common forms of VHD in patients with CA, namely aortic stenosis (AS), mitral regurgitation (MR), and tricuspid regurgitation (TR). We will discuss our current understanding on the epidemiology, disease mechanisms, echocardiographic features, clinical implications, and therapeutic options of these conditions. For this review, we searched relevant studies in PubMed/Medline (updated April 2023) using the following terms: *amyloidosis*, *cardiac amyloidosis*, *valve*, *valvular*, *stenosis*, and *regurgitation*. Given the design of this work as a narrative review, no formal criteria for study selection or appraisal were enforced.

## Aortic stenosis

### Epidemiology

AS is the most common form of VHD in the general population, and its prevalence increases with age [7]. CA and AS both cause left ventricular (LV) wall thickening and diastolic dysfunction up to a restrictive phenotype [8]. The coexistence of AS and CA is being increasingly recognized (Table 1). In 2017, Castaño et al. reported a prevalence of ATTR-CA of 16% among 156 patients with severe AS planned to undergo transcatheter aortic valve replacement (TAVR). Patients with ATTR-CA had thicker interventricular septum (IVS), higher LV mass index, and lower stroke volume index as well as more advanced diastolic and systolic dysfunction [9]. Similarly, Scully et al. reported that approximately 1 in 7 patients currently undergoing TAVR has occult CA [10]. In elderly patients with AS undergoing surgical aortic valve replacement (SAVR), a screening with cardiac magnetic resonance (CMR) and intraoperative biopsies were performed on 146 individuals with severe AS referred to SAVR. Six of 146 patients with calcific AS aged >65 years had ATTR-CA (prevalence 5.6%) [11]. In another study, ^99m^Tc-diphosphono-propanodicarboxylic acid (DPD) scintigraphy was performed in patients with degenerative AS scheduled to undergo valve replacement (either SAVR or TAVR) when one of the following echocardiographic red flags was present: increased thickness of atrioventricular valves, interatrial septum or right ventricular (RV) free wall, pericardial effusion, and myocardial granular sparkling. Five of the 43 AS patients showed at least one red flag and underwent scintigraphy that displayed myocardial uptake in all 5 cases. Endomyocardial biopsy consistently revealed amyloid infiltration by ATTR amyloid [12]. In a more recent study, 46 Indian patients aged ≥65 years undergoing SAVR were screened with ^99m^Tc-PYP scintigraphy, which showed a significant myocardial uptake (i.e., with a heart-to-contralateral-lung [H:CL] uptake ≥1.50) in 3/32 (9.4%) cases. The basal septum was biopsied intra-operatively in scintigraphy-positive cases and was unexpectedly negative in all cases. On the contrary, 33 valves (72%) showed amyloid deposits, with ATTR amyloid in 19, and the other cases simply labelled as “ATTR-negative” [13]. These results may suggest that amyloid may deposit selectively in valve structures, but methodological issues with myocardial biopsy sampling and/or histology may play a role, and further evidence is needed. There is indeed evidence that amyloid deposits may be detected in the LV also in the context of AS, revealing that the dual disease (AS and CA) is not uncommon [11].

**Table 1:** Prevalence of cardiac amyloidosis in patients with aortic stenosis

First author, year (ref)	Population characteristics	Diagnostic technique	Prevalence of CA
Castaño et al., 2017 [9]	156 patients with severe AS planned to receive TAVR	^99m^Tc-PYP cardiac scintigraphy	16% (all ATTR-CA)
Nietlispach et al., 2012 [67]	20 AS patients with previous TAVR	Autopsy or histology after surgery	33% (no data on amyloidosis type)
Treibel et al., 2016 [11]	146 elderly (<65 years old) patients with AS undergoing SAVR	CMR and intraoperative biopsies	5.6% (all ATTR-CA)
Longhi et al., 2016 [12]	43 patients with degenerative AS undergoing SAVR or TAVR	^99m^Tc-DPD scintigraphy and endomyocardial biopsy	11.6% (all ATTR-CA)
Cavalcante et al., 2017 [68]	113 AS patients	CMR	8% (all ATTR-CA)
Singal et al., 2021 [13]	46 Indian patients ≥65 years undergoing SAVR	^99m^Tc-PYP and basal interventricular septum biopsy	9.4% (all ATTR-CA)

*AL* amyloid light chain, *AS* aortic stenosis, *ATTR* amyloid transthyretin amyloidosis, *CA* cardiac amyloidosis, *CMR* cardiac magnetic resonance, *DPD* diphosphono-propanodicarboxylic acid, *LGE* late-gadolinium enhancement, *SAVR* surgical aortic valve replacement, *TAVR* transcatheter aortic valve replacement, *Tc-PYP* technetium pyrophosphate

Up to 15% of patients with AS and up to 30% of those with low-flow low-gradient AS have CA [14]. ATTR-CA is more often associated with AS than AL-CA, and the coexistence of AS and CA is more common in elderly people and males [15]. Although several studies have investigated the prevalence of ATTR-CA among subjects with AS, evidence about AL-CA is still limited. For example, two studies enrolling patients scheduled for TAVR reported a prevalence of 14–16% for ATTR-CA but did not complete the diagnostic flow chart despite the presence of monoclonal proteins in the serum of approximately 3% of participants [9, 10]. Two studies including 191 and 407 patients referred for TAVR have identified a single case of isolated AL-CA in both cohorts [16, 17]. Therefore, the prevalence of AL-CA in patients with AS seems low, but it may be underestimated.

### Possible disease mechanisms

**Fig. 1:**
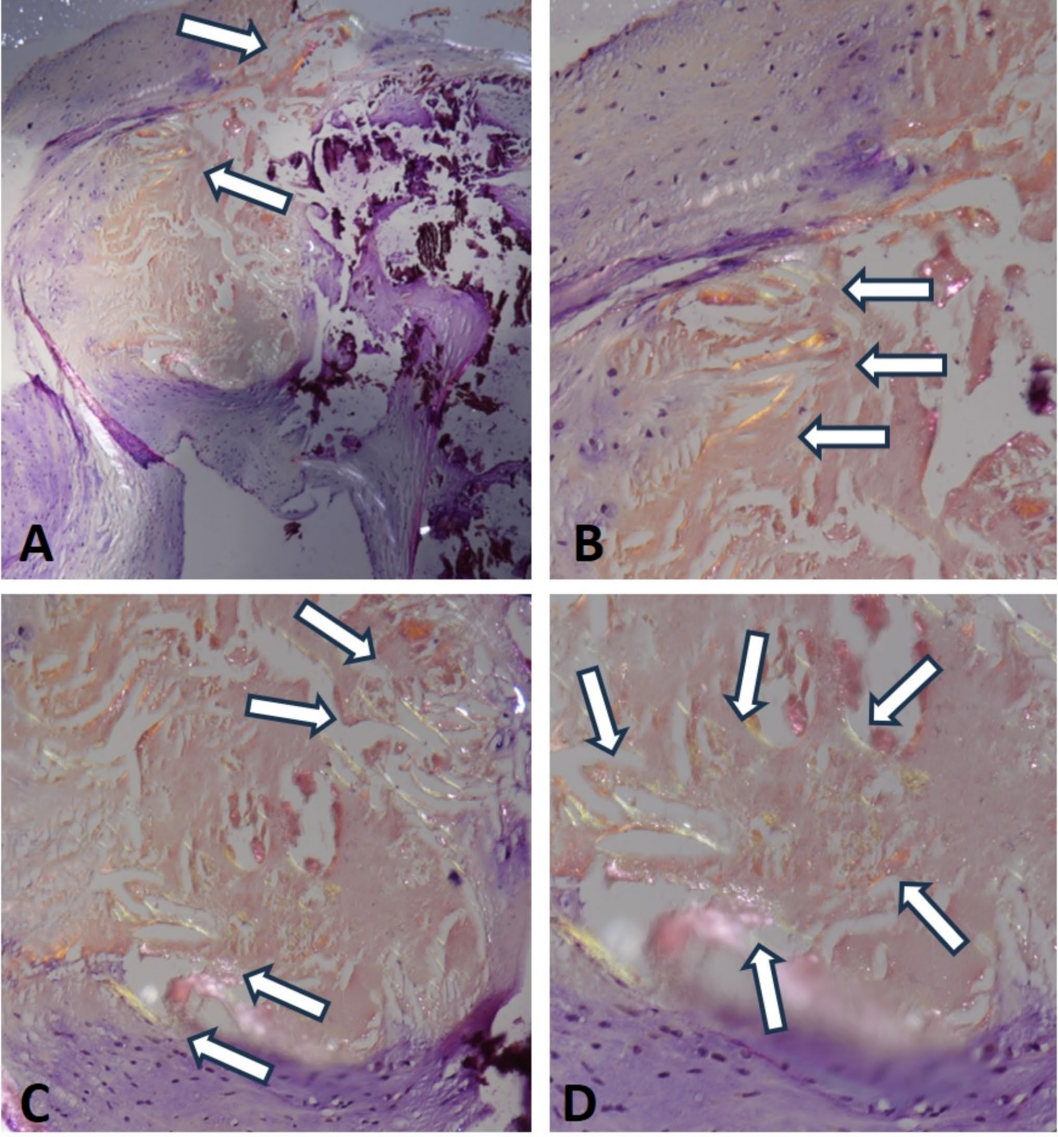
Amyloid and calcium deposits in a patient with transthyretin amyloidosis undergoing aortic valve surgery for severe stenosis. Calcifications of an aortic cusp are associated to amyloid deposits that are shown by green bi-refringence by Congo red staining under polarized light (within the circle). Original magnification: ×4 (**A** and **B**) and ×10 (**C** and **D**)

**Fig. 2:**
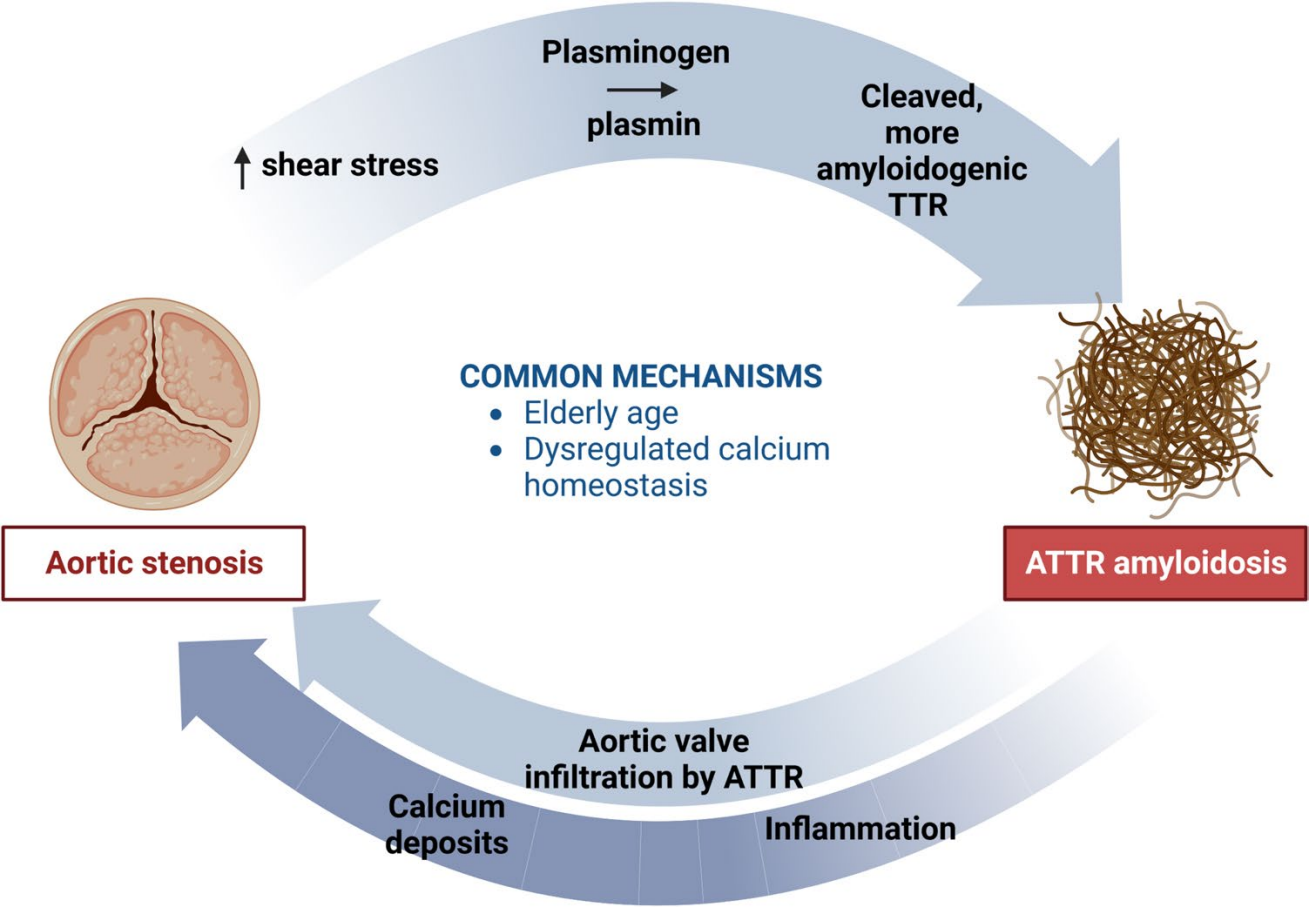
Possible mechanisms linking amyloid transthyretin cardiac amyloidosis (ATTR-CA) and aortic stenosis (AS). See text for further details

The exact mechanism linking AS and (ATTR-)CA is currently unknown. Current evidence suggests that the pathophysiological mechanisms of the two conditions may co-exist in a bidirectional relationship, thus influencing each other in a sort of vicious cycle. Firstly, amyloid precursors may deposit in valve tissues by diffusion through the valve endothelium or (in patients with AS) through small vessels [18] and be retained in valve tissues because of the absence of lymphatic vessels. On the other side, increased shear stress in case of AS could also promote plasminogen activation to plasmin, which would then cleave the TTR tetramer into more amyloidogenic species [18]. Furthermore, the interaction between calcium ions and TTR tetramers may increase protein susceptibility to proteolytic cleavage, and then amyloid deposition, among patients with calcific AS [19]. On histological analysis, amyloid and calcium deposits are spatially associated (Fig. 1). A bidirectional relationship between amyloid and calcium deposits might be hypothesized, with oxidation and calcification causing amyloid deposition in the valves and, conversely, amyloid deposition leading to inflammation and valve mineralization, thus exacerbating valvular disease [20] (Fig. 2). However, the interplay between AS and CA remains elusive as the amount of calcium in the aortic cusps of patients with AS-CA is lower than in lone AS, as discussed below. The age-related degeneration of tissue homeostasis is an additional condition usually shared by CA and AS, which both affect elderly patients.

### Diagnosis

**Fig. 3:**
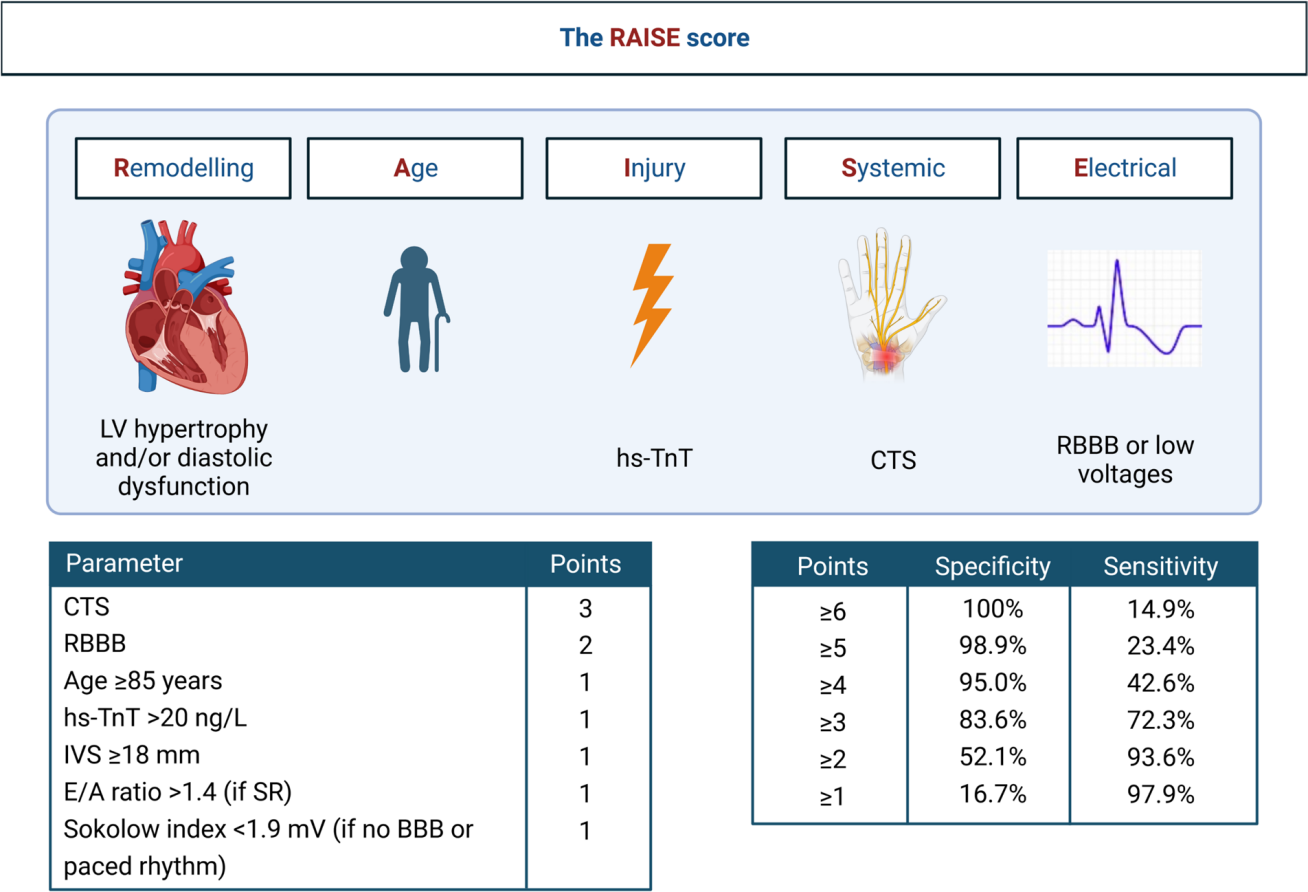
A diagnostic score for cardiac amyloidosis in patients with aortic stenosis. The elements of the RAISE score are reported: carpal tunnel syndrome (CTS), right bundle branch block (RBBB), high-sensitivity troponin T (hs-TnT) > 20 ng/L, interventricular septal thickness (IVS) ≥ 18 mm, E/A ratio > 1.4 (if in sinus rhythm [SR]), and Sokolow index < 1.9 mV (if no BBB or paced rhythm is present). The sensitivity and specificity of different score values are reported. From Nitsche et al. [17]

ATTR-CA should be suspected in every case of AS, and particularly in elderly male patients with a clinical history of carpal tunnel syndrome, lumbar spinal stenosis, and spontaneous tendon ruptures, as well as premature pacemaker implantation, disproportionate HF symptoms despite non-severe AS, and indicators of RV failure. AL-CA should be suspected in patients with light chain monoclonal disorders, often associated with nephropathy (proteinuria), autonomic dysfunction, polyneuropathy, macroglossia, and spontaneous bruising. Electrocardiogram findings in CA patients include Q waves without a history of myocardial infarction, low voltages despite increased LV wall thickness, and conduction abnormalities [21, 22]. Laboratory red flags include chronically raised troponin and disproportionally elevated N-terminal pro-B-type natriuretic peptide (NT-proBNP) levels compared to the degree of LV dysfunction [23]; in AL-CA, pathological free light chains, serum, and/or urine immunofixation are key parameters for an early diagnosis and treatment. The echocardiogram may show severe biventricular wall thickening, myocardial granular sparkling, and severe LV longitudinal systolic dysfunction with apical sparing. In CA, CMR typically shows a characteristic pattern of circumferential subendocardial late-gadolinium enhancement (LGE) coupled with elevated native T1 and (extracellular volume) ECV values [24]. When any one of the red flags above is spotted in AS patients, CA should be suspected, and the standard diagnostic algorithm should be followed [25]. To help clinicians diagnose CA, Nitsche et al. have developed a score including the stronger predictors of CA [17] (Fig. 3).

The clinical usefulness of left atrial and ventricular strain analysis in the diagnosis of CA in AS has been recently reported. In patients with moderate to severe AS, the peak longitudinal strain rate in the left atrium together with the relative apical longitudinal strain index may be a good predictor of ATTR-CA [26]. Evidence suggests that global longitudinal strain (GLS) is decreased in both ATTR-CA and AS, but the reduction is more pronounced in case of dual disease [27]. A cut-off value of −14% has been proposed to detect ATTR-CA in subjects with AS, although poorly specific [27]. Furthermore, although apical sparing is a common finding in ATTR-CA, it is also frequent in lone AS; thus, it should be considered in combination with other clinical parameters [27, 28].

Recently, ECV measurement by computed tomography (CT) has gained popularity as a tool to diagnose CA in patients with AS, which is of utmost clinical usefulness considering that almost all AS patients undergo contrast CT for planning surgical/percutaneous interventions. ECV values are markedly increased in patients with CA following amyloid accumulation [29, 30], and ECV measurement could aid in the differential diagnosis between CA-AS and AS alone. A CT-ECV threshold of 31.4% was shown to have a sensitivity of 94% and a specificity of 48% for ATTR-CA in a cohort of elderly patients with severe AS [31]. Patients with ATTR-CA were found to have significantly higher septal CT-ECV values than those with AL-CA. CT-ECV may then become a screening tool for ATTR-CA in the AS population and also help differentiate ATTR-CA from AL-CA [32, 33].

Low-flow low-gradient AS is commonly found in CA. In cases of discordant AS grading, European guidelines recommend confirming AS severity by using dobutamine stress echocardiography and/or aortic valve calcium score by CT [34]. A threshold calcium score ≥2000 Hounsfield units (HU) in males and ≥1200 HU in females may identify with a high likelihood severe AS; on the contrary, AS is unlikely with a score of <1600 HU in males and <800 HU in females [34]. Nonetheless, patients with CA may have a lower relative aortic valve calcium score versus those without CA [35]. A retrospective analysis on patients with AS-CA or lone AS found significantly lower calcium scores in CA-AS [36]. Aortic valve calcium threshold scores might therefore not always be reliable because of the risk of underestimating severe AS in case of CA. An ongoing multicenter Italian study (the “Calcium score values of stenotic aortic valves in patients with and without cardiac amyloidosis” [CAUSATIVE] study) will define the optimal values of valve calcification to diagnose CA in patients with AS. Dobutamine stress echocardiography may be useful to confirm the presence of true severe AS in patients with CA [37]. However, further studies are needed.

### Treatment and outcome

The coexistence of CA and AS seems to be associated with a worse prognosis than CA or AS alone. A meta-analysis on 4243 patients from 21 studies showed that patients who had both AS and CA had a higher mortality rate than those with lone AS or CA [38]. Similarly, in a systematic review of 4 observational studies, CA-AS was associated with a twofold increase in all-cause mortality compared with lone AS [39]. Another meta-analysis on 17 studies and 1988 AS patients provided similar results. In comparison to 1220 patients with AS alone, 178 patients with CA-AS had higher mortality (odds ratio [OR] 2.25, *p* = 0.004) [40]. Two ongoing studies, ATTRact-AS (NCT03029026) and AMYLOCARTESIAN (NCT02260466) are trying to elucidate the prevalence and prognostic impact of CA in elderly people with AS.

Most patients with CA-AS are not candidate to SAVR because of their age and comorbidities. In a study by Nitsche et al., most patients (82%) received TAVR as planned while 16% received medical management, and only 3% underwent SAVR. TAVR increased survival compared to medical therapy. Patient survival following TAVR was similar between CA-AS and lone AS [17]. A meta-analysis confirmed that TAVR improves survival in patients with CA-AS compared to medical treatment. The risk for stroke, vascular complications, life-threatening hemorrhage, acute renal injury, and 30-day mortality did not differ significantly between patients with CA-AS (*n* = 75) vs. those with lone AS (*n* = 536), with comparable risks of stroke, vascular complications, life-threatening bleeding, acute kidney injury, and 30-day mortality, with just a trend towards an increased risk of permanent pacemaker implantation (*p* = 0.085) [41]. Another study reported a similar rate of complications in AS-CA vs. lone AS (*p* = 0.77), with no deaths during a 30-day follow-up [42]. Finally, a meta-analysis found that the risk of death after TAVR did not differ significantly between patients with AS-CA vs. those with lone AS [43].

Long-term follow-up data after TAVR are limited. A study on 120 patients undergoing TAVR followed for 1 year revealed that those with ATTR-CA (*n* = 25) remained more symptomatic, had higher NT-proBNP, and had no regression in LV mass [44]. Current evidence is limited to ATTR-CA, while little is known about AL-CA, which still holds a poor prognosis and only rarely can undergo TAVR.

Based on the latest recommendations from the European Society of Cardiology (ESC), the optimal follow-up for patients with CA should not differ in presence of AS, and *vice versa* [45]. However, given the higher frailty and increased likelihood of developing heart failure, CA patients undergoing interventional procedures such as TAVR should be monitored with particular care.

## Mitral regurgitation

### Epidemiology

Limited data are available on the prevalence of CA in patients with MR, or MR in patients with CA (Table 2). A multicenter prospective study on 120 patients undergoing transcatheter edge-to-edge repair (TEER) for MR reported a prevalence of CA of 11.7% (10% ATTR-CA), and early amyloid infiltration (Perugini grade 1) in 7.5% [46]. Mitral valve disease is often associated with aortic valve disease. An echocardiographic study on 150 patients with AL-CA found mitral and/or aortic valve thickening (>3 mm) in 42% of patients, isolated mitral valve thickening in 30 (48%), isolated aortic thickening in 11 (17%), and thickening of both valves in 22 (35%). Patients with mitral and/or aortic valve thickening were older and had a higher NYHA class than the other patients, and several signs of greater cardiac remodeling (e.g., higher LV wall thickness and mass, higher E/e′ ratio and systolic pulmonary artery pressure) [47]. A histological analysis of 150 surgically resected heart valve specimens (AS, *n* = 100; MR, *n* = 24; other aortic or mitral diseases, *n* = 26) found amyloid deposits in 55% of samples, with the highest prevalence in AS (74%), and lower in MR (29%) [48]. The high prevalence of amyloid deposits was in agreement with an autopsy series showing that 38 out of 75 (51%) and 21 out of 61 (34%) sclerotic or sclerocalcific lesions of the aortic valves and mitral valves, respectively, had amyloid deposits [49]. However, as in case of CA, the isolated presence of amyloid deposits within the valve provides an uncertain clinical significance. Indeed, valvular amyloidosis includes the coexistence of amyloid aggregates with both structural and functional alterations [50].

**Table 2:** Studies assessing the coexistence of cardiac amyloidosis and mitral or tricuspid valve regurgitation

First author, year (ref)	Population characteristics	Diagnostic technique	CA prevalence
Mohty et al., 2017 [47]	63 patients with AL-CA	Transthoracic echocardiography	Mitral disease: 43% Mitral and aortic disease: 35%
Kristen et al., 2010 [48]	150 surgically resected heart valve specimens	Histology	Amyloid in 29% of valves explanted for MR (no data on amyloidosis type)
Yokota et al., 1994 [49]	61 cases of sclerotic or sclerocalcific lesions of the mitral valve	Histology	Amyloid deposits in 34% of mitral valves (amyloid type not characterised)
Donà et al., 2022 [46]	120 patients undergoing TEER of the mitral valve	Standard diagnostic workup	CA in 11.7% (86% ATTR-CA, 7% AL-CA, 7% mixed)
Xu et al., 2019 [69]	7733 mitral valves surgically removed over 10 years	Histology	CA in 0.2% (93% ATTR-CA, 7% not specified)
Fagot et al., 2021 [60]	283 patients with ATTRwt- or AL-CA	Transthoracic echocardiography	26% with moderate-to-severe TR (65% ATTR-CA, 35% AL-CA)
Nemes et al., 2022 [61]	27 CA patients	2D and 3D echocardiography	Alterations of the tricuspid annulus in 100% (78% AL-CA and 22% ATTR-CA)

*AL* amyloid light chain, *ATTR* (wt) (wild-type) amyloid transthyretin, *CA* cardiac amyloidosis, *LVT* left heart valve thickening, *TEER* transcatheter edge-to-edge repair, *TR* tricuspid regurgitation

### Pathophysiology and imaging features

**Fig. 4:**
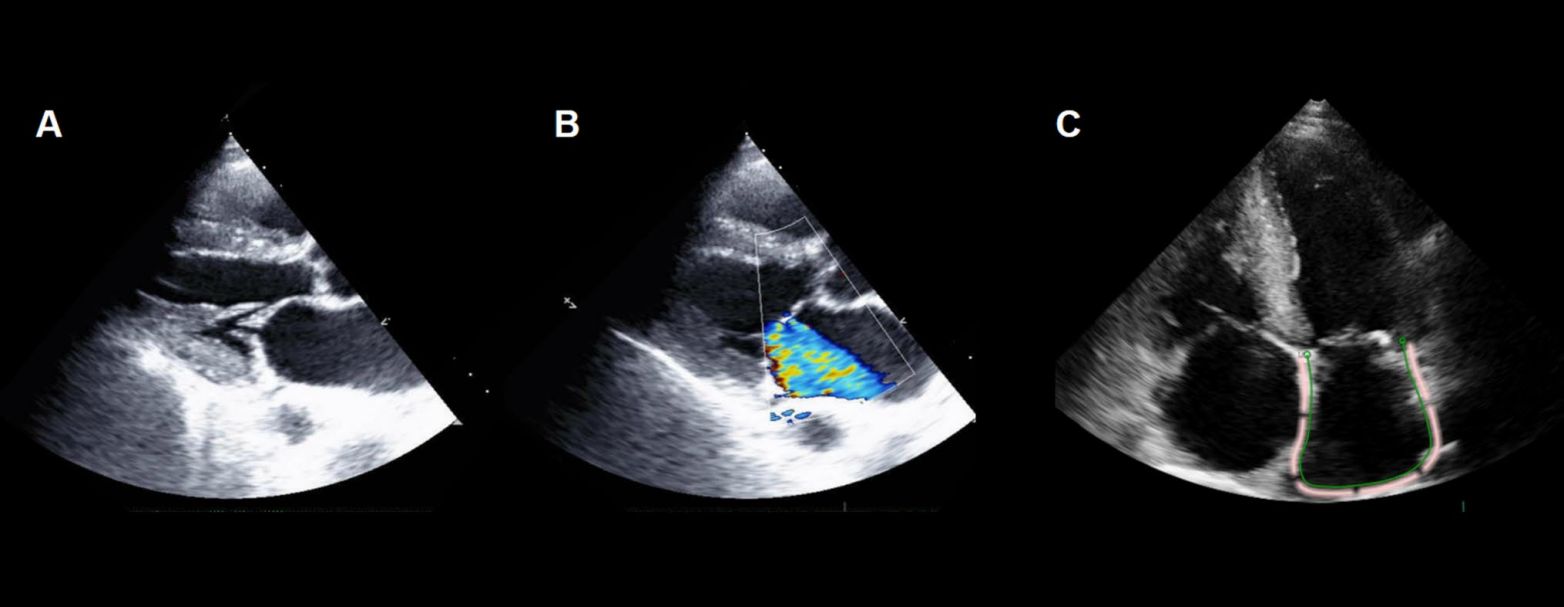
Echocardiographic features of mitral regurgitation (MR). Features of MR in a 50-year-old man with amyloid light-chain cardiac amyloidosis: elongated and thickened leaflets and subvalvular apparatus (**A**), massive regurgitation (**B**), left atrial turbulence with reduced function (peak atrial longitudinal strain 5%)

**Fig. 5:**
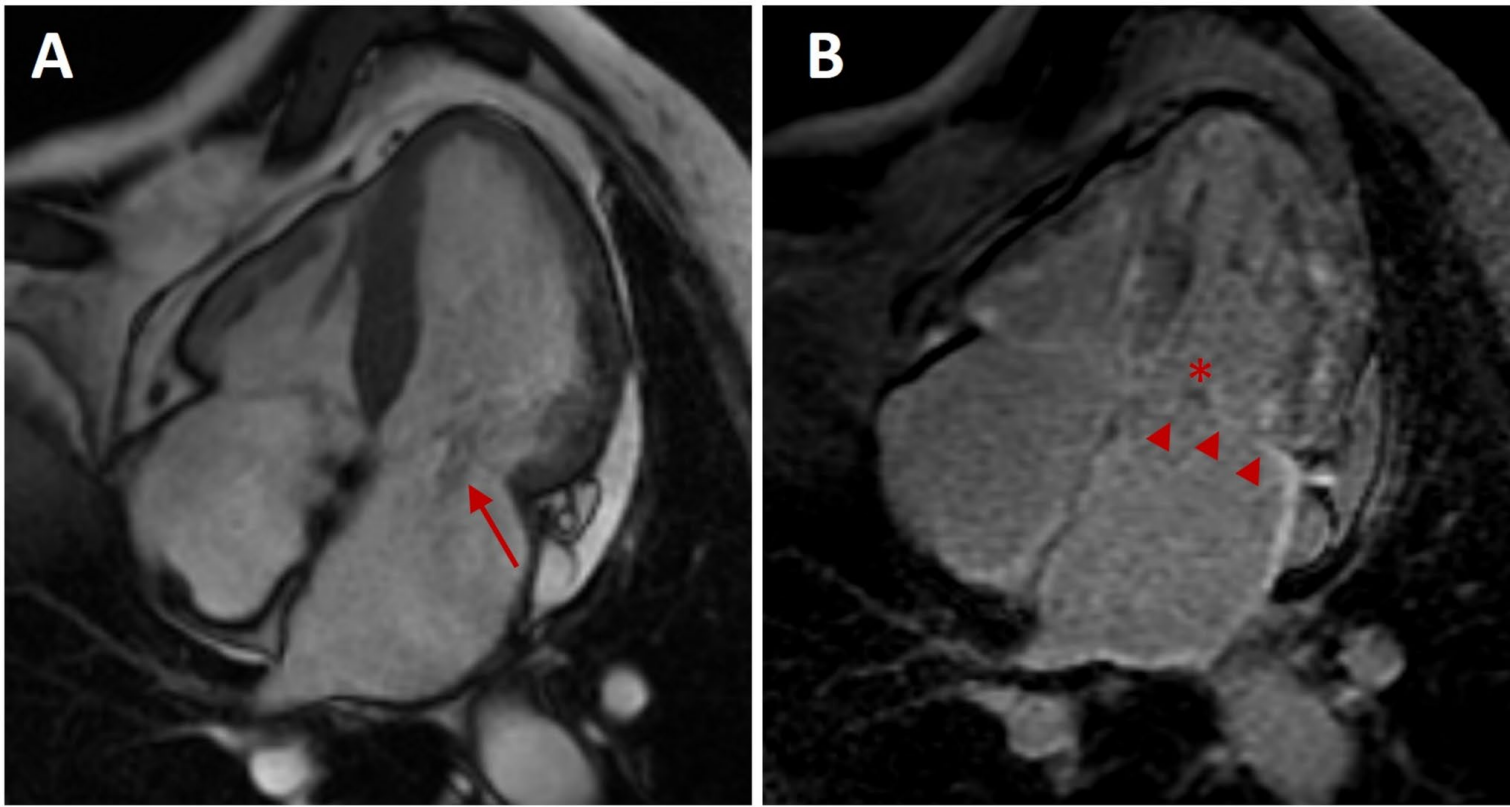
Magnetic resonance imaging in a AL-CA patient with moderate mitral regurgitation. **A** Cine steady-state free-precession four-chamber view in early systole, showing an increased biventricular and biatrial wall thickness, with flow turbulence (*red arrow*) due to mitral regurgitation. **B** Late gadolinium four-chamber view, showing biventricular and biatrial enhancement consistent with amyloid deposition, more prominent in the subendocardim; note late enhancement of the mitral leaflets (*red arrowheads*), with some very small hypointense areas due to calcifications (*asterisk*)

MR in CA is a multifactorial process caused by different structural and functional alterations. Amyloid deposition within the valve, anulus, and subvalvular structures causes leaflet thickening and retraction, thus resulting in MR (Carpentier class III) (Fig. 4). The typical layered arrangement of leaflets is significantly impaired, with increased fibrous tissue in the fibrosa, an increased amount of glycosaminoglycans in the spongy layer, and changes to the central core of loose connective tissue. The flexibility and sliding motion of the layers are also impaired. On macroscopic examination, the typical scallop segmentation is lost, and both leaflets appear stiff and swollen. The geometry of the mitral annulus is altered [51], the commissures are stretched, and chordae tendinae are thickened and shortened. Chordal rupture and leaflet prolapse have also been reported [52]. In our experience, patients with ATTR-CA display thickened mitral chordae tendineae and a shortened/hidden/restricted posterior leaflet more often than those with AL-CA [53], although leaflet calcifications can be found even in patients with AL-CA (Fig. 5). In a large cohort of patients with ATTR-CA (*n* = 877), Chacko et al. reported a progressive worsening of structural and functional parameters (i.e., LV systolic and diastolic function, deformation-based analysis, and right heart structure and function), with a more rapid deterioration in patients with V122I ATTR-CA [54], in agreement with previously reported data [55]. Amyloid deposits extend to the fibrous annulus and in the surrounding atrial and ventricular myocardium, which stiffen and alter the entire valvular apparatus [54]. Indeed, amyloid infiltration leads to loss of normal atrial architecture, with pathological remodeling of both vessels and myocardium. The increased atrial stiffness leads to a reduction of the reservoir and contractile functions, with subsequent increased atrial pressure [56]. Interestingly, left atrium remodeling aligns with that typical of restrictive cardiomyopathies (namely concentric remodeling with non-compliant chambers) and differs from that commonly found in isolated MR, where atrium enlargement is a predominant feature [57]. Furthermore, echocardiographic analysis revealed that CA is associated with significant mitral anulus dilation, stiffness and functional impairment [51, 54], thus supporting the low prevalence of mitral stenosis. Finally, given the different pathophysiological mechanisms in CA-caused MR, it is likely that classical echocardiographic parameters may not be sufficient or accurate. To this purpose, further studies are needed in order to identify specific cut-offs and parameters to assess MR severity in patients with CA.

#### Prognosis and treatment

Outcome data are scarce, but MR and its progression seem associated with worse outcome. In the study by Chacko et al., the only variables linked with survival were a worsening in the degree of MR and TR at 12 and 24 months, and MR remained an independent risk factor after adjusting for established predictors [54]. In the study by Mohty et al., patients with mitral and/or aortic valve thickening had also a greater risk of all-cause death after adjusting for age, sex, NYHA class, and LV ejection fraction (hazard ratio 1.90; 95% confidence interval 1.10–3.34; *p* = 0.02). No data were reported on mitral valve thickening alone [47]. The most plausible reason of the prognostic value of MR is the decreased forward flow, which is particularly relevant given that the LV is small and its diastolic relaxation is impaired.

Volz et al. reported the results of an initial experience on five CA patients undergoing percutaneous mitral valve repair (PMVR) for moderate to severe MR non-randomly compared to a small control group of seven similar CA patients with MR without intervention. The success of the PMVR procedure was 100%, and the residual MR was still mild to moderate after 12 months. Survival differences between the PMVR group and the control group suggested that the procedure might improve patient outcome. However, no significant improvements were seen in the echocardiography parameters of systolic and diastolic function at 1-year follow-up [58]. Only two case reports of mitral valve replacement are currently available, with a total of 6 cases treated with PMVR. Four out of 6 had ATTR-CA and 2 had AL-CA. Positive procedural outcomes were reported in all 6 patients in terms of survival and improved NYHA class at 6 and 12 months [59]. In a study by Donà et al., CA-MR and lone MR showed a similar rate of procedural success and periprocedural complications. Over a median follow-up of 1.7 years, patients with CA-MR (12 ATTR, 1 AL, and 1 combined ATTR/AL) had a 2.5fold higher risk of hospitalization for HF, but similar mortality than those with lone MR [46]. Overall, further evidence is needed on the feasibility and results of PMVR in patients with CA, and the criteria for patient selection to avoid futility.

## Tricuspid regurgitation

**Fig. 6:**
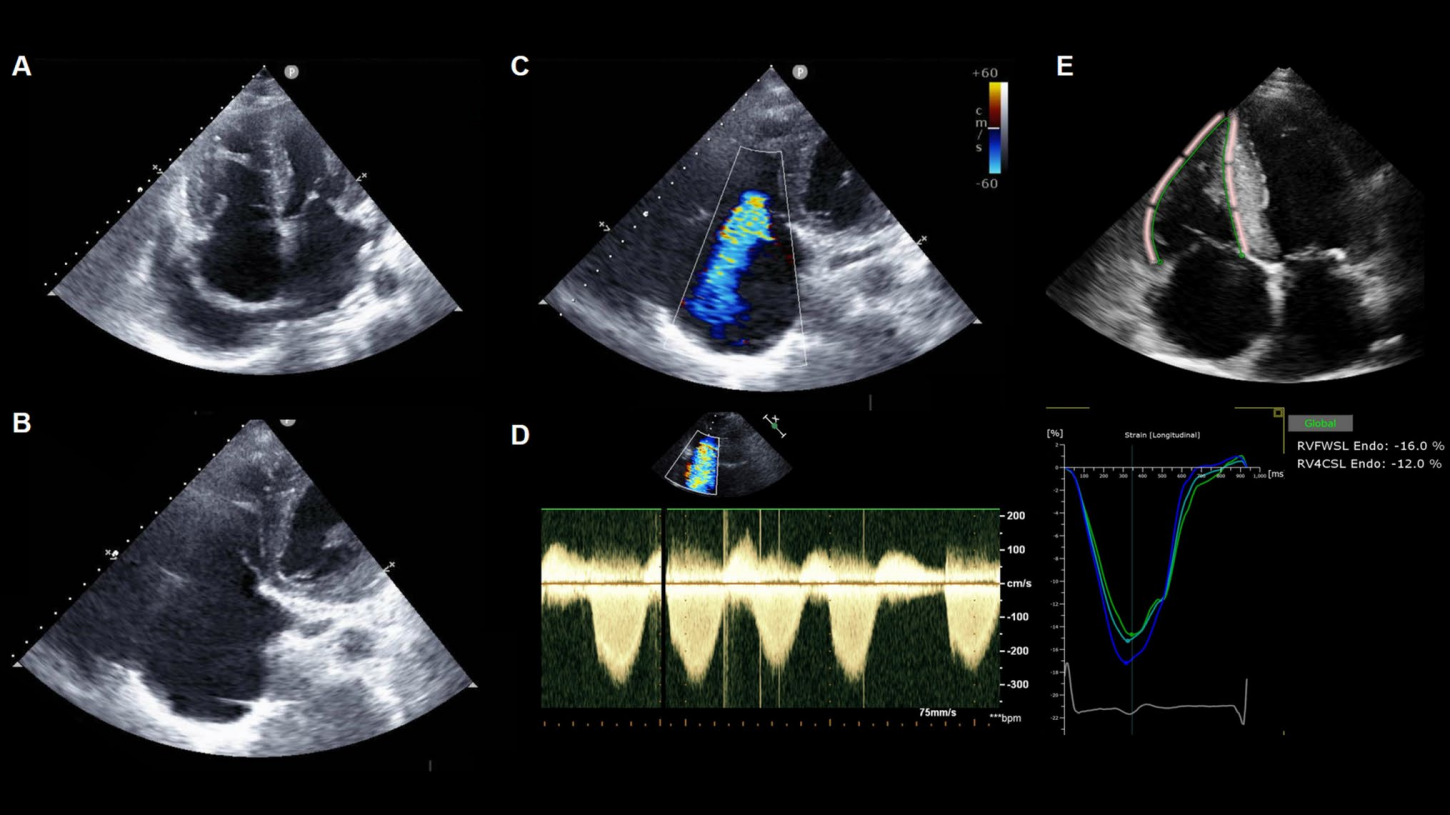
Echocardiographic features of tricuspid regurgitation (TR). Features of TR in an 83-year-old woman with amyloid transthyretin cardiac amyloidosis: severely enlarged right ventricle (RV) and right atrium with retracted septal leaflet (**A, B**); pericardial effusion (**B**); massive regurgitation (**C**, **D**); depressed systolic RV function (RV free wall strain −16%)

**Fig. 7:**
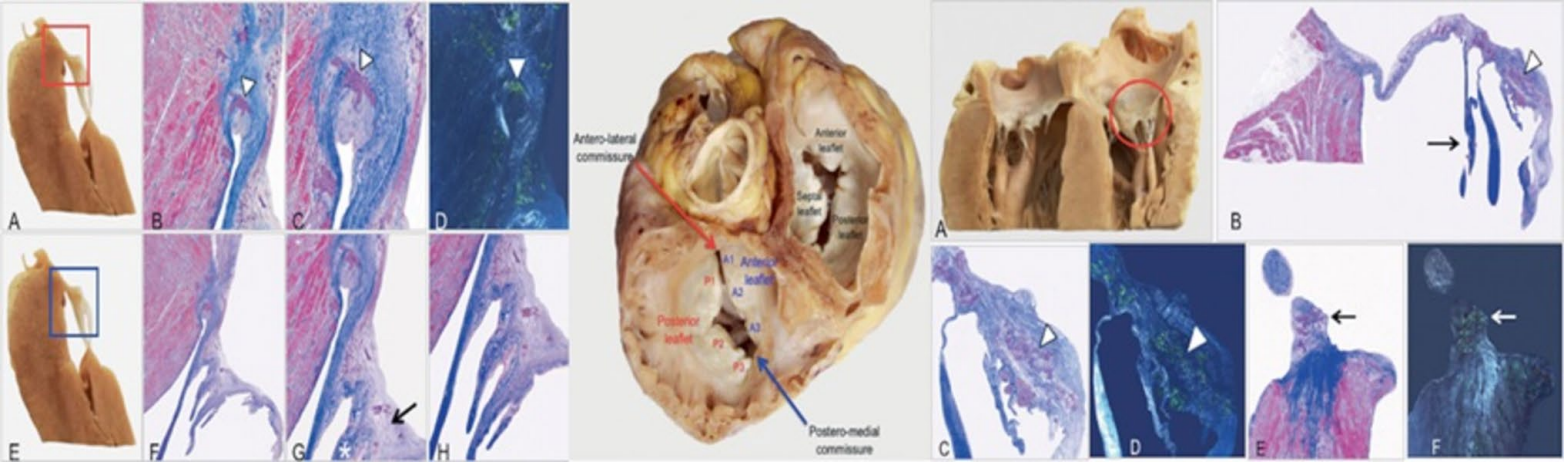
Histological features of mitral and tricuspid infiltration. Histological sections from the native heart of a 44-year-old lady with hereditary transthyretin amyloid cardiomyopathy (Ser23Asn variant). Left panel: Mitral valve posterior leaflet. **A**–**D** Amyloid deposits within the fibrous annulus (arrowheads). A Macroscopic specimen; **B** Azan Mallory (AM) trichrome, original magnification (OM) ×25; **C** AM trichome, OM ×50; **D** Congo red (CR) staining, OM ×50. **E**–**H** Fibrosis is increased and distributed along the ventricular side (**G**, asterisk); loose connective tissue rich in glycosaminoglycans evident in the atrial side (**G**, arrow). Macroscopic specimen; **F** scanned slide; **G** AM trichrome, OM ×25; **H** AM trichrome, OM ×25. Middle panel: macroscopic view of atrio-ventricular valves with stiffened annulus, thickened leaflets, loss of normal scallop segmentation. Right panel: **A** Macroscopic specimen of the anterior leaflet of the tricuspid valve, which appears swollen and stiffened, due to numerous nodular amyloid deposits (**B**–**D**) (arrowheads) and fibrous tissue. Tendinous cords are markedly thickened (**B**, arrow). **E**, **F** The apex of papillary muscle indicates fibrosis in blue and multiple amyloid deposits (arrows). **B** Scanned slide; **C** AM trichrome, OM ×25; **D** CR staining, OM ×25; AM trichrome, OM ×25; **F** CR staining, OM ×25.hide. Reprinted with permission from Chacko et al. [54] **Central Illustration** Main manifestations of valve involvement in patients with cardiac amyloidosis (CA) AL, amyloid light-chain; AS, aortic stenosis; ATTR, amyloid transthyretin; CT, computed tomography; ECV, extracellular volume; MR, mitral regurgitation; MV, mitral valve; PMVR, percutaneous mitral valve replacement; TAVR, transcatheter aortic valve replacement; TR, tricuspid regurgitation; TVR, tricuspid valve replacement

Data on tricuspid valve involvement in CA are very limited (Table 2). In a cohort of 283 patients with CA (61% ATTRwt and 39% AL), 26% displayed moderate-to-severe TR [60]. TR in CA seems often due to tricuspid annular dilation, RV dilation, and dysfunction (Fig. 6). A small study reported that patients with CA (*n* = 27) had dilated end-diastolic and end-systolic tricuspid annulus diameter, area, and perimeter. Valve morphology is generally more altered in ATTR-CA [61]. Histological features of mitral and tricuspid infiltration can be found in Fig. 7 [54].

TR has a major prognostic impact in patients with ATTRwt-CA, but not in those with AL-CA [60]. This difference is partly explained by the fact that AL-CA patients typically have a worse prognosis than ATTR-CA. In AL-CA, the systemic disease may therefore progress more rapidly than TR and its hemodynamic and prognostic consequences [60]. In a recent study by Tomasoni et al., right ventricular to pulmonary artery coupling (measured as tricuspid annular plane systolic excursion/pulmonary artery systolic pressure [TAPSE/PASP] ratio) was a strong and independent predictor of outcome in patients with CA [62]. A TAPSE/PASP ratio <0.45 mm/mmHg independently predicted all-cause death or HF hospitalization in both ATTR- and AL-CA with a twofold increase in risk in the patients with lower values. Interestingly, the TAPSE/PASP ratio was more effective in predicting prognosis than TAPSE or PASP alone [62]. In a recent study, 8 patients who successfully underwent transcatheter tricuspid valve replacement for severe to torrential TR ATTRwt-CA were compared to 21 patients without CA. Device success and rates of hospitalization or death were similar compared with those in the control group at 3 months. TR reduction in CA patients was less extensive than that in control group, and CA patients showed no significant improvement of structural right heart parameters. Nonetheless, percutaneous repair led to an improvement in NYHA class and 6-minute walking distance even in CA patients [63].

## Conclusions and future perspectives

Valvular disease is being increasingly recognized as a common feature of CA, and an important determinant of symptoms (Central Illustration). Most notably, AS is now listed among red flags for CA [45], and atrioventricular valve thickening has been included among echocardiographic red flags in an echocardiographic screening [64]. Valvular involvement imposes a further hemodynamic load on stiffened cardiac chambers, first the LV but also the left atrium and right heart chambers. The hemodynamic effects of valvular disease and their relationship with exercise capacity could be optimally assessed through exercise echocardiography or the combination of cardiopulmonary exercise testing and echocardiography, when patients are still able to undergo this testing. The same techniques could help assess the acute effects of drugs acting on the preload (e.g., diuretics) or the afterload (e.g., ACE-inhibitors) and investigate whether disease-modifying therapies have positive effects over valve function through their effects on amyloid deposition in the LV and possibly also in cardiac valves. Promising results have come from studies testing the possibility to induce amyloid clearance through monoclonal antibodies [65, 66]. The pharmacological-mediated reduction of amyloid burden within the valvular tissue may determine beneficial effects as well as the regression of valvular disease. The current gold standard therapy for ATTR amyloidosis (namely TTR stabilizers and gene silencers) slows down disease progression. Further studies should investigate whether the treatment of patients with early CA could be helpful to prevent severe forms of amyloid-related valvular disease. We need also more data on the prognostic impact of valvular involvement in patients with CA, on the possible differences between AL and ATTR and on the identification of the optimal timing for intervention. Finally, we need a better understanding of the pathophysiology of valve disease (most notably aortic valve disease), looking for possible therapeutic targets.

## Data Availability

Not applicable.
